# Impact of hepatic inflammation and fibrosis on the recurrence and long-term survival of hepatitis B virus-related hepatocellular carcinoma patients after hepatectomy

**DOI:** 10.1186/s12885-024-12187-9

**Published:** 2024-04-15

**Authors:** Xiangyong Hao, Liangliang Xu, Xiang Lan, Bo Li, Hui Cai

**Affiliations:** 1 Division of Liver Surgery, Department of General Surgery, West China Hospital, Sichuan University, 610041 Chengdu, China; 2 Department of General Surgery, Gansu Provincial Hospital, 730000 Lanzhou, China; 3 Department of Hepatobiliary Surgery, The First Affiliated Hospital, Chongqing Medical University, 400016 Chongqing, China

**Keywords:** Hepatic inflammation, Hepatic fibrosis, HBV, Hepatectomy, Prognosis

## Abstract

**Background:**

Underlying liver disease is correlated with hepatocellular carcinoma (HCC) development in patients with hepatitis B virus (HBV) infection. However, the impact of hepatic inflammation and fibrosis on the patients’ prognoses remains unclear.

**Methods:**

The clinicopathological data of 638 HBV-infected patients with early-stage HCC between 2017 and 2019 were prospectively collected. Hepatic inflammation and fibrosis were evaluated by experienced pathologists using the Scheuer score system. Survival analysis was analyzed using the Kaplan–Meier analysis.

**Results:**

Application of the Scheuer scoring system revealed that 50 (7.9%), 274 (42.9%), and 314 (49.2%) patients had minor, intermediate, and severe hepatic inflammation, respectively, and 125 (15.6%), 150 (23.5%), and 363 (56.9%) patients had minor fibrosis, advanced fibrosis, and cirrhosis, respectively. Patients with severe hepatitis tended to have a higher rate of HBeAg positivity, higher HBV-DNA load, elevated alanine aminotransferase (ALT) levels, and a lower proportion of capsule invasion (all P*p* < 0.05). There were no significant differences in the recurrence-free and overall survival among the three groups (*P* = 0.52 and *P* = 0.66, respectively). Patients with advanced fibrosis or cirrhosis had a higher proportion of HBeAg positivity and thrombocytopenia, higher FIB-4, and larger tumor size compared to those with minor fibrosis (all *P* < 0.05). Patients with minor, advanced fibrosis, and cirrhosis had similar prognoses after hepatectomy (*P* = 0.48 and *P* = 0.70). The multivariate analysis results indicated that neither hepatic inflammation nor fibrosis was an independent predictor associated with prognosis.

**Conclusions:**

For HBV-related HCC patients receiving antiviral therapy, hepatic inflammation and fibrosis had little impact on the post-hepatectomy prognosis.

## Introduction

Hepatocellular carcinoma (HCC) is one of the most prevalent cancers worldwide, and viral hepatitis is the most common cause of cirrhosis and HCC in Asia and Africa [1]. Epidemiological evidence suggests that approximately 10–25% of chronic HBV infections lead to HCC. China accounts for approximately half of the HCC cases reported worldwide due to the high prevalence of HBV infection [2]. Radical surgical resection is the mainstay treatment for patients with resectable HCCs, including some HCC patients with intermediate-stage disease [3]. Nevertheless, HCC is an aggressive cancer and is characterized by a high recurrence post-hepatectomy. A previous study suggested that 30–50% and up to 70% of patients suffered from recurrence within 2 and 5 years, respectively, making recurrence the major cause of mortality [4]. Unlike other solid tumors, HCC frequently occurs with underlying liver disease. Patients with underlying liver cirrhosis were at a 20-fold risk of developing HCC. Michael et al. concluded that underlying liver disease played a critical role in the prognosis of viral-related HCC [5]. To elucidate the impact of the underlying liver disease on the prognosis of patients with HCC was beneficial for HCC management.

Antiviral drugs, such as tenofovir and entecavir, were widely recommended for patients with HBV infection. Sustained virological response could effectively achieve histological improvement and thus reduce and prolong HCC development and improve prognosis [6, 7]. A liver assaulted by HBV can rapidly produce an immune reaction and lead to massive inflammatory cell infiltration. With chronic and persistent hepatitis, liver fibrosis and even cirrhosis can occur. The Scheuer scoring was first proposed by PJ Scheuer in 1991 and has since been widely used to evaluate hepatic inflammation and fibrosis in patients with chronic viral hepatitis [8]. This scoring system can precisely classify the impaired liver into different degrees of inflammation or fibrosis.

Inflammation is known to be closely related to tumor development and prognosis. Numerous studies have reported that systemic inflammation has a negative impact on the prognosis of patients with HCC [9]. Regarding the correlation between hepatitis and prognosis, Xiang et al. reported that worse hepatitis was associated with worse prognosis among patients with non-cirrhotic HBV-associated HCC [10]. Using a nonalcoholic steatohepatitis (NASH) mouse model, researchers demonstrated that blockade of hepatocyte inflammation could prevent the development of HCC [11]. Since antiviral therapy is widely utilized, the relationship between hepatitis and the prognosis of patients with HCC needs further investigation in a real-world study.

Fibrosis and cirrhosis contribute to HCC development [12]. The negative impact of cirrhosis on postoperative complications is well known [13]. While the impact of cirrhosis on the long-term survival of patients with HCC remained inconsistent. Some studies reported that the long-term prognosis of patients with HCC and cirrhosis was worse than that of those without cirrhosis [14, 15]. However, high-quality studies have reported no significant difference in the prognosis between HCC patients with cirrhosis and without cirrhosis [4, 16, 17]. Among HCC patients without cirrhosis, Xiang et al. reported that a worse prognosis was associated with advanced fibrosis [10]. Hence, whether the degree of fibrosis, including cirrhosis, impacted the prognosis of HBV-related patients with HCC who were receiving antiviral therapy needs to be investigated.

Herein, we retrospectively enrolled HBV-related patients and attempted to elucidate the impact of pathological hepatic inflammation and fibrosis based on the Scheuer score system on the prognosis of patients with early-stage HCC after hepatectomy.

## Methods and materials

Data from 638 HCC patients who underwent hepatectomy between December 2017 and January 2019 in the Department of Liver Surgery and Liver Transplantation Center, West China Hospital, were retrospectively analyzed. The inclusion criteria were: (1) patients with pathologically proven HCC; (2) patients who had undergone hepatectomy; (3) those with HBsAg positivity; (4) those receiving antiviral therapy(15 patients for adefovir, 4 patients for lamivudine, 530 patients for enticave, 75 patients for tenofovir, 9 patients for telbivudine, and 5 patients switching from others to enticave); (5) those with BCLC stage A disease; and (6) patients with normal renal function. The exclusion criteria were: (1) presence of recurrent HCC; (2) positive surgical margin; (3) history of HCV; (4) co-current cancers; and (5) incomplete clinicopathological information or follow-up data. The following data were prospectively collected preoperatively: demographic features, routine blood tests; liver function tests; HBV infection status; HBV-DNA load; alpha-fetoprotein (AFP) level; tumor size, number, and differentiation; and status of microvascular invasion (MVI). This study protocol was approved by the Ethics Committee of West China Hospital. The requirement for informed consent was waived due to the retrospective nature of the study.

### Evaluation of hepatic inflammation and fibrosis

The degrees of hepatitis and hepatic fibrosis were evaluated using the Scheuer scoring system [8] as follows inflammation: G0, no portal or periportal and lobular necro-inflammatory activity; G1, portal or periportal inflammation and minimal occasionally spotty lobular inflammation; G2, mild piecemeal portal or periportal necrosis and mild or focal lobular necrosis; G3, moderate piecemeal portal or periportal necrosis and moderate or noticeable hepatocellular change inside liver lobule; and G4, severe piecemeal portal or peri FIB-4 scoring portal necrosis and severe or diffuse hepatocellular damage inside the lobule. Fibrosis: S0, absence of fibrosis; S1, fibrous portal expansion; S2, periportal or rare portal-portal septa; S3, fibrous septa with architectural distortion; and S4, cirrhosis). Minor, intermediate, and severe hepatic inflammation were defined as G1, G2, and G3-G4, respectively. Minor fibrosis, advanced fibrosis, and cirrhosis were defined as S1-S2, S3, and S4, respectively [18].

### Follows up

The follow-up plan was the same as that reported in a previous study from our center. All patients were followed up by the outpatient investigations department or phone interview postoperatively. Routine blood tests, liver function tests, serum AFP levels, HBV-DNA, and radiological examinations, including ultrasound, contrast-enhanced ultrasound, contrast-enhanced CT, or MRI, were performed at each investigation. Antiviral drugs, such as entecavir or tenofovir, were administered based on the guidelines. Postoperative HCC recurrence was defined as the presence of two typical imaging findings or one positive imaging finding plus increased AFP levels or a positive histological finding. Re-treatment methods, including liver transplantation, repeat surgery, radiofrequency ablation (RFA), transarterial chemoembolization (TACE), sorafenib, and best supportive care, were routinely recommended by our multiple disciplinary team (MDT), which mainly comprised hepatic surgeons, oncologist, and radiologist, based on the recurrence pattern and functional liver reserve. Overall survival (OS) time was defined as the interval between the operation and death or the last follow-up. Recurrence-free survival (RFS) time was defined as the time interval between the operation and the first detectable recurrence. The final follow-up visit occurred at the end of January 2022 or until death.

### Statistical analysis

Categorical data were displayed as numbers (%) and were compared using the χ2 test or Fisher’s exact test. Continuous variables were expressed as means ± standard deviations (SD) and were compared using the t-test. Univariate and multivariate analyses were performed using the Cox proportional hazards model. Potential risk factors with *P* < 0.05 in the univariate analysis were included in the multivariate analysis model using the step-forward method. Survival analysis was performed using the Kaplan–Meier analysis and was compared using the log-rank test. Statistical analysis was performed using IBM SPSS statistics software (version 20.0) for Windows. A *P* value < 0.05 in two-tailed tests was considered statistically significant.

## Results

### Patient characteristics

Clinicopathological features of 638 HBV-related HCC patients are displayed in Table 1. There were 538 (84.3%) male patients, 134 (21.0%) patients with HBeAg positivity, 339 (53.1%) patients with high HBV-DNA load (> 2000 IU/mL), and 262(41.1%) patients with an AFP level of > 400 ng/mL. The average tumor diameter was 4.5 cm. Twelve (1.9%) patients had multiple tumors, 169 (26.5%) presented with MVI, and 60 (9.4%) with satellite lesions. The tumor capsule was invaded in 268 (42.0%) patients. There were 190 (29.8%) patients with thrombocytopenia, 22 (3.4%) with hypoproteinemia, 19 (3.0%) with hyperbilirubinemia, and 262 (41.1%) with elevated ALT levels.

Based on the G score (Scheuer scoring system), 50 (7.9%), 274 (42.9%), and 314 (49.2%) patients were divided into minor, intermediate, and severe hepatitis groups. Patients with severe hepatitis had a higher proportion of HBeAg positivity (26.4% vs. 16.0% vs. 11.2%), a higher proportion of thrombocytopenia, a higher proportion of MVI, and higher ALT levels than those with minor and intermediate hepatitis. The other variables were not significantly different between the three groups(Table 1).

**Table 1:** Characteristic stratified by hepatic inflammation

	minor	intermediate	severe	*P* value
	*n* = 50	*n* = 274	*n* = 314	
Gender (male, %)	43 (86.0)	226 (82.5)	269 (85.7)	0.538
Age, y	50.00 [40.25, 60.50]	50.00 [45.00, 58.75]	51.00 [45.00, 60.00]	0.642
AFP (> 400 ng/mL, %)	16 (32.0)	112 (40.9)	134 (42.7)	0.361
HBeAg (%)	13 (26.0)	41 (15.0)	80 (25.5)	0.005
Tumor size, cm	4.85 [3.00, 7.32]	4.05 [3.00, 7.50]	4.50 [3.00, 6.80]	0.995
Multiple tumor (%)	1 (2.0)	2 (0.7)	9 (2.9)	0.163
Poorly differentiation, (%)	25 (50.0)	124 (45.3)	151 (48.1)	0.717
Hepatic fibrosis				< 0.001
minor	18 (36.0)	84 (30.7)	23 (7.3)	
advanced	12 (24.0)	62 (22.6)	76 (24.2)	
cirrhosis	20 (40.0)	128 (46.7)	215 (68.5)	
MVI, (%)	8 (16.0)	69 (25.2)	92 (29.3)	0.114
Satellite, (%)	2 (4.0)	21 (7.7)	37 (11.8)	0.092
Fatty liver, (%)	12 (24.0)	52 (19.0)	80 (25.5)	0.165
Capsule invasion, (%)	26 (52.0)	124 (45.3)	118 (37.6)	0.056
HBV-DNA, (> 2000 IU/mL, %)	19 (38.0)	130 (47.4)	190 (60.5)	0.001
PT, s	12.10 [11.70, 12.50]	12.10 [11.50, 12.90]	12.10 [11.60, 12.88]	0.881
INR	1.00 [1.00, 1.10]	1.00 [1.00, 1.10]	1.00 [1.00, 1.10]	0.773
RBC, x10^9^/L	4.65 [4.26, 5.16]	4.74 [4.30, 5.04]	4.73 [4.35, 5.10]	0.661
HB, g/L	144.50 [125.50, 154.00]	145.00 [132.00, 155.00]	147.00 [136.00, 155.75]	0.127
PLR(> 101, %)	14 (28.0)	122 (44.5)	110 (35.0)	0.017
NLR(> 2.6, %)	15 (30.0)	94 (34.3)	95 (30.3)	0.548
ALT(> 40 IU/L, %)	16 (32.0)	100 (36.5)	146 (46.5)	0.019
AST(> 40 IU/L, %)	21 (42.0)	92 (33.6)	132 (42.0)	0.094
ALB(≤ 40 g/L, %)	1 (2.0)	7 (2.6)	14 (4.5)	0.38
TBIL(> 28 umol/L, %)	2 (4.0)	5 (1.8)	12 (3.8)	0.33
PLT(≤ 100 × 10^9^/L, %)	17 (34.0)	66 (24.1)	107 (34.1)	0.024
FIB-4(> 3.6, %)	17 (34.0)	66 (24.1)	97 (30.9)	0.12

AFP: alpha-fetoprotein, HBeAg: hepatitis B virus e antigen, MVI: microvascular invasion, TBIL: total bilirubin, ALT: alanine transferase, AST: Aspartate aminotransferase, ALB: albumin, PLT: platelet, PT: prothrombin time, INR: international normalized ratio, RBC, red blood cell, HB: hemoglobin, PLR: platelet to lymphocyte ratio, NLR: neutrophil to lymphocyte ratio, FIB-4: fibrosis index based on the four factors

Based on the S score (Scheuer scoring system), 125 (15.6%), 150 (23.5%), and 363 (56.9%) patients were divided into minor fibrosis, advanced fibrosis, and cirrhosis groups. Patients with cirrhosis had a higher proportion of HBeAg positivity (26.4% vs. 16.0% vs. 11.2%), a higher proportion of thrombocytopenia (38.3% vs. 21.3% vs. 15.2%), a higher proportion of FIB-4 (> 3.6: 33.3% vs. 22.0% vs. 10.8%), and lower proportion of PLR > 101.1(33.1% vs. 40.7% vs. 52.0%), and smaller tumor diameter (4.0 cm vs. 4.75 cm vs. 5.8 cm) compared to those with minor and advanced fibrosis; however, they had normal ALT levels (36.9% vs. 38.7% vs. 42.4%). The other variables were not significantly different between the three groups(Table 2).

**Table 2:** characteristic stratified by hepatic fibrosis

	minor	advanced	cirrhosis	*P* value
	*n* = 125	*n* = 150	*n* = 363	
Gender(male, %)	105 (84.0)	130 (86.7)	303 (83.5)	0.659
Age, y	51.00 [44.00, 61.00]	51.00 [45.00, 61.00]	51.00 [45.00, 58.00]	0.844
AFP (> 400 ng/mL, %)	46 (36.8)	54 (36.0)	162 (44.6)	0.109
HBeAg (%)	14 (11.2)	24 (16.0)	96 (26.4)	< 0.001
Tumor size, cm	5.80 [4.00, 11.00]	4.75 [3.00, 7.50]	4.00 [2.70, 6.20]	< 0.001
Multiple tumor (%)	1 (0.8)	1 (0.7)	10 (2.8)	0.174
Poorly differentiation, (%)	56 (44.8)	66 (44.0)	178 (49.0)	0.499
G stratification, (%)				< 0.001
minor	18 (14.4)	12 (8.0)	20 (5.5)	
intermediate	84 (67.2)	62 (41.3)	128 (35.3)	
severe	23 (18.4)	76 (50.7)	215 (59.2)	
MVI, (%)	36 (28.8)	33 (22.0)	100 (27.5)	0.349
satellite, (%)	11 (8.8)	16 (10.7)	33 (9.1)	0.829
fatty liver, (%)	28 (22.4)	40 (26.7)	76 (20.9)	0.368
capsule invasion, (%)	56 (44.8)	72 (48.0)	140 (38.6)	0.112
HBV-DNA, (> 2000IU/mL, %)	74 (59.2)	75 (50.0)	190 (52.3)	0.282
PT, s	11.90 [11.30, 12.40]	12.00 [11.40, 12.80]	12.20 [11.70, 13.00]	< 0.001
INR	1.00 [1.00, 1.10]	1.00 [1.00, 1.10]	1.10 [1.00, 1.10]	0.005
RBC, x10^9^/L	4.75 [4.27, 5.11]	4.69 [4.34, 5.01]	4.75 [4.34, 5.12]	0.702
HB, g/L	143.00 [132.00, 154.00]	145.00 [134.00, 155.75]	147.00 [133.50, 155.00]	0.288
PLR(> 101, %)	65 (52.0)	61 (40.7)	120 (33.1)	0.001
NLR(> 2.6, %)	46 (36.8)	53 (35.3)	105 (28.9)	0.16
ALT(> 40 IU/L, %)	51 (40.8)	74 (49.3)	137 (37.7)	0.052
AST(> 40 IU/L, %)	53 (42.4)	58 (38.7)	134 (36.9)	0.552
ALB(≤ 40 g/L, %)	4 (3.2)	8 (5.3)	10 (2.8)	0.342
TBIL(> 28 umol/L, %)	0 (0.0)	6 (4.0)	13 (3.6)	0.089
PLT(≤ 100 × 10^9^/L, %)	19 (15.2)	32 (21.3)	139 (38.3)	< 0.001
FIB-4(> 3.6, %)	26 (20.8)	33 (22.0)	121 (33.3)	0.004

### Survival analysis

**Fig. 1:**
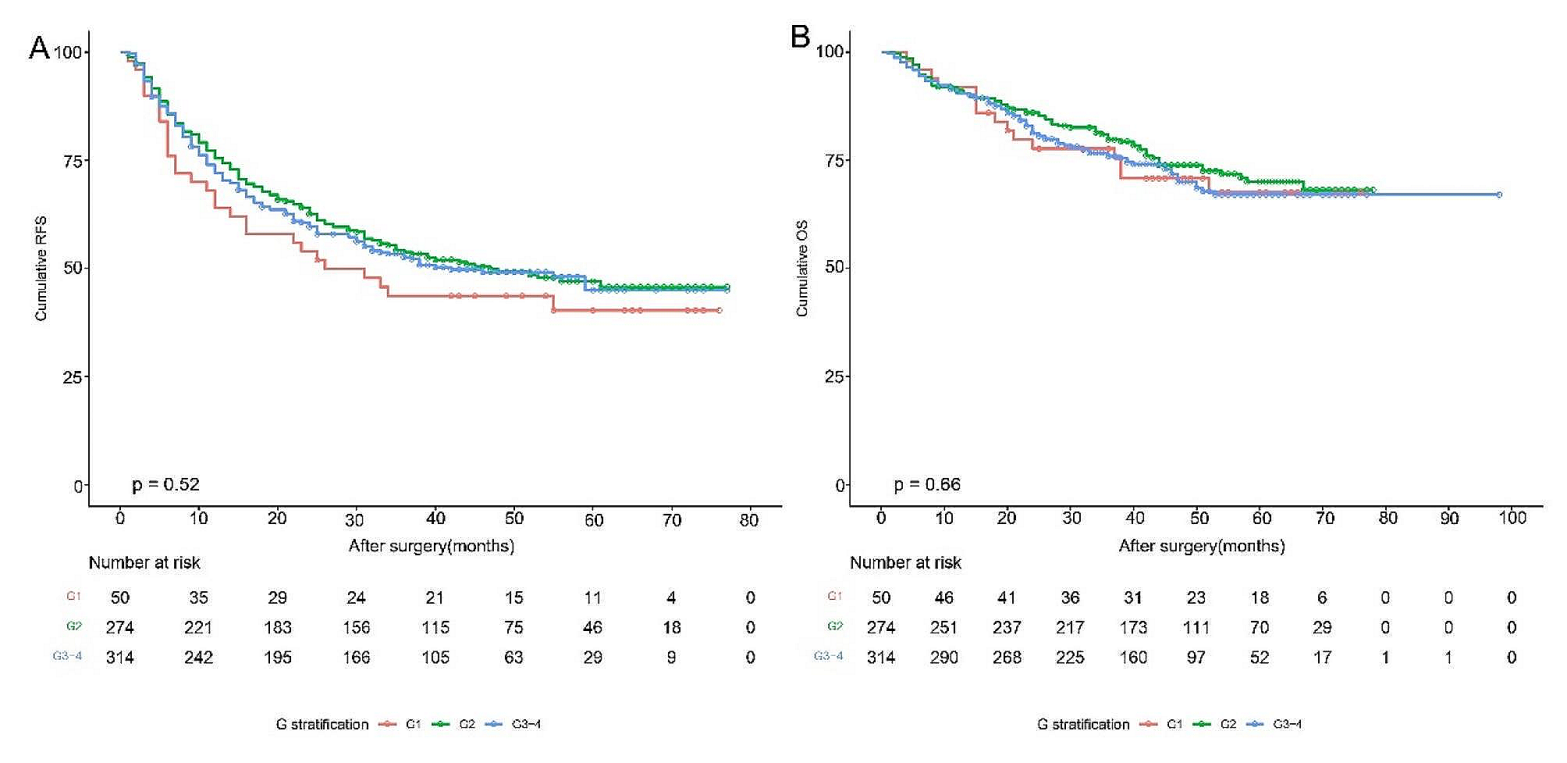
Kaplan-meier analysis for patients with minor, intermediate and severe hepatic inflammation. The three groups had similar RFS (A) and OS (B).RFS: Recurrence free survival; OS: overall survival

The 1-year, 3-year, and 5-year RFS rates of patients with minor, intermediate, and severe hepatitis were 64.0%, 75.5%, and 72.0%; 43.7%, 53.8%, and 52.6%; and 33.7%, 40.3%, and 45.0%, respectively (*P* = 0.52). The OS rates of patients with severe hepatitis were similar to that of patients with minor or intermediate hepatitis at 1 year (92.0%, 91.2%, and 90.4%), 3 years (77.7%, 79.8%, and 76.0%) and 5 years (67.6%, 70.1%, and 67.1%) (*P* = 0.66)( Fig. 1).

**Fig. 2:**
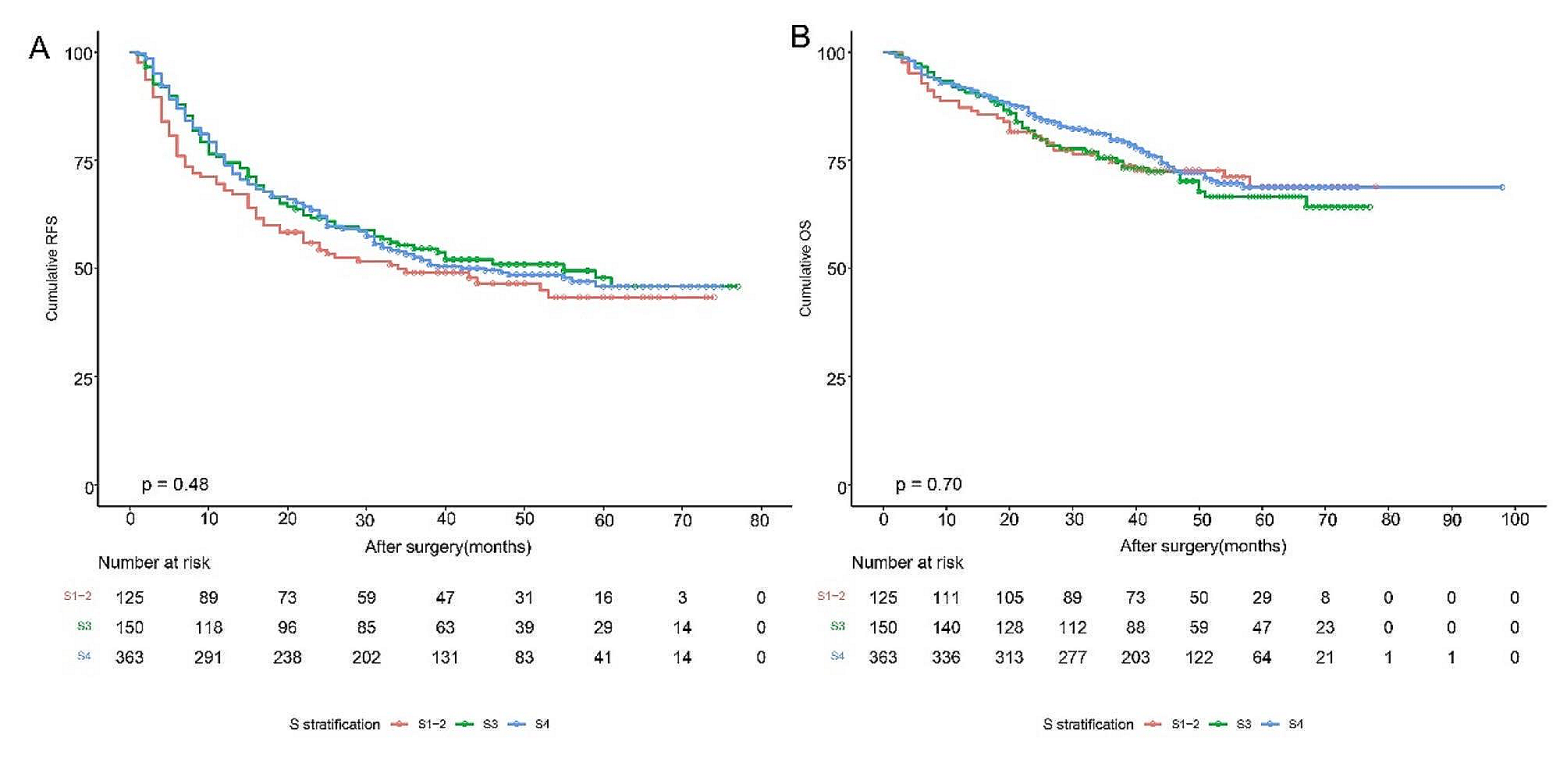
Kaplan-meier analysis for patients with minor, advanced fibrosis and cirrhosis. The three groups had similar RFS (A) and OS (B)

The 1-year, 3-year, and 5-year RFS rates of patients with minor fibrosis, advanced fibrosis, and cirrhosis were 68.0%, 74.5%, and 73.9%; 49.0, 54.6%, and 52.6%; 43.3%, 47.9%, and 45.9%, respectively (*P* = 0.48). The OS rates of patients with cirrhosis were similar to that of patients with minor and advanced fibrosis at 1-year (87.2%, 91.3%, and 92.0%), 3-years (74.7%, 75.6%, and 79.7%), and 5-years (68.9%, 66.6%, and 68.8%), respectively, (*P* = 0.70)(Fig. 2).

### Risk factors related to RFS and OS

The results of the univariate analysis showed that HBeAg positivity; tumor size, number, and differentiation; MVI; satellite lesions; capsule invasion; high HBV-DNA load, PT levels, AST levels, AFP (> 400 ng/ml) levels, and NLR levels were significant factors related to higher cumulative risk of HCC recurrence. All these factors were included in the multivariate analysis. The results of multivariate analysis suggested that HBeAg positivity (HR = 1.312, 95% confidence interval [CI]:1.039–1.658, *P* = 0.023), tumor size (HR = 1.076, 95% CI:1.047–1.107, *P* < 0.001), tumor number (HR = 1.508, 95% CI:1.191–1.910, *P* = 0.001), MVI (HR = 1.446, 95% CI:1.154–1.813, *P* = 0.001), satellite lesions (HR = 2.195, 95% CI:1.663–2.899), capsule invasion (HR = 1.315, 95% CI:1.065–1.632, *P* = 0.011), and elevated AST level (HR = 1.286, 95% CI:1.037–1.595, *P* = 0.022) were independent risk factors of HCC recurrence (Table 3).

**Table 3:** Univariate and multivariable analyses to identify risk factors of RFS

	HR	95%CI	*p* value	HR	95%CI	*P* value
Gender	1.42	1.017–1.973	0.039			
Age	0.99	0.982–1.002	0.105			
AFP	1.40	1.125–1.747	0.003			
HBeAg	1.31	1.018–1.689	0.036	1.671	1.293–1.001	0.049
Tumor size	1.13	1.098–1.154	< 0.001	1.111	1.078–1.046	< 0.001
Number	1.29	0.638–2.596	0.482			
Poorly differentiation	1.47	1.179–1.826	0.001			
G stratification	0.96	0.810–1.147	0.68			
S stratification	0.94	0.818–1.077	0.368			
MVI	1.93	1.528–2.439	< 0.001	1.709	1.330–1.036	0.025
satellite	2.90	2.146–3.929	< 0.001	2.913	2.129–1.557	< 0.001
Fatty liver	0.75	0.567–0.984	0.038			
Capsule invasion	1.71	1.374–2.128	< 0.001	1.764	1.400-1.111	0.004
HBV-DNA	1.14	0.914–1.417	0.246			
PT	1.14	1.018–1.267	0.023			
INR	2.12	0.654–6.845	0.211			
RBC	1.12	0.940–1.346	0.200			
HB	1.00	0.992–1.005	0.704			
PLR	1.21	0.966–1.508	0.097			
NLR	1.33	1.056–1.668	0.015			
ALT	1.15	0.921–1.432	0.220			
AST	1.74	1.398–2.167	< 0.001	1.657	1.305–1.028	0.029
ALB	1.37	0.801–2.339	0.250			
TBIL	1.03	0.549–1.936	0.924			
PLT	1.08	0.854–1.369	0.518			
FIB-4	1.28	1.010–1.618	0.041			

The results of the univariate analysis showed that HBeAg positivity, high HBV-DNA load, tumor size, tumor differentiation, MVI, satellite lesions, capsule invasion, PT, Hb, and elevated ALT, AST, NLR, and AFP levels (> 400 ng/ml) were significant predictors of OS. All these factors were included in the multivariate analysis. The results of multivariate analysis suggested that tumor size (HR = 1.080, 95% CI:1.043–1.118, *P* < 0.001), tumor differentiation (HR = 1.345, 95% CI:1.010–1.789, *P* = 0.042), MVI (HR = 1.756, 95% CI:1.304–2.365, *P* < 0.001), satellite lesions (HR = 2.350, 95% CI:1.682–3.284), PT (HR = 1.151, 95% CI:1.005–1.317, *P* = 0.042), AFP (HR = 1.497, 95% CI:1.138–1.971, *P* = 0.004), and elevated AST (HR = 1.513, 95% CI:1.128–2.028, *P* = 0.006) were independent risk factors of OS. Moreover, hepatic inflammation and cirrhosis were not independent risk predictors(Table 4).

**Table 4:** Univariate and multivariable analyses to identify risk factors of OS

Variables	HR	95%CI	*p* value	HR	95%CI	*P* value
Gender	1.39	0.880–2.190	0.159			
Age	0.99	0.979–1.006	0.289			
AFP	2.05	1.521–2.763	< 0.001	1.61	1.194–2.182	0.002
HBeAg	1.39	0.994–1.940	0.054			
Tumor size	1.15	1.116–1.186	< 0.001	1.08	1.044–1.127	< 0.001
Number	0.53	0.131–2.135	0.371			
Poorly differentiation	1.85	1.371–2.507	< 0.001			
G stratification	1.06	0.838–1.346	0.620			
S stratification	0.94	0.784–1.135	0.537			
MVI	2.60	1.912–3.534	< 0.001	1.52	1.096–2.124	0.012
Satellite lesion	3.30	2.282–4.765	< 0.001	2.23	1.521–3.283	< 0.001
Fatty liver	0.73	0.495–1.066	0.103			
Capsule	1.88	1.394–2.532	< 0.001	1.38	1.016–1.899	0.039
HBV-DNA	1.59	1.173–2.162	0.002			
PT	1.22	1.056–1.417	0.007			
INR	5.28	1.117–24.941	0.036			
RBC	1.08	0.848–1.385	0.523			
HB	0.99	0.985–1.002	0.140			
PLR	1.39	1.031–1.874	0.031			
NLR	1.68	1.246–2.277	0.001			
ALT	1.48	1.101–1.993	0.009			
AST	2.41	1.788–3.249	< 0.001	1.70	1.230–2.351	0.001
ALB	1.32	0.650–2.688	0.440			
TBIL	0.55	0.176–1.725	0.306			
PLT	0.98	0.712–1.357	0.916			
FIB-4	1.56	1.143–2.116	0.005			

## Discussion

Hepatic inflammation and fibrosis usually occur in patients with chronic HBV infection. Consistent hepatic inflammation and fibrosis were closely related to the incidence and prognosis of HCC. The role of hepatic inflammation and fibrosis in the post-hepatectomy prognosis of patients needs to be clarified. In the present study, 638 consecutive patients with HBV-related early-stage HCC who received radical hepatectomy were enrolled. All patients received antiviral therapy at the time of surgery, and antiviral therapy was modulated during follow-up periods if virological suppression was not achieved. Unfortunately, the majority of patients with early-stage HCC had intermediate (43.0%) or severe (49.2%) hepatitis. Approximately half of the patients with early-stage HCC (56.9%) have pathological cirrhosis. This suggests that histological impairment is challengeable for HBV-related patients. More attention should be paid to the management of underlying liver disease. The result of the survival analysis indicated that patients with minor, intermediate, and severe hepatitis at the time of surgery had a similar prognosis in terms of RFS and OS. Meanwhile, patients with minor fibrosis, advanced fibrosis, and cirrhosis at the time of surgery had comparable prognoses. To further exclude confounding factors, a Cox proportional hazards regression analysis was performed. We consistently found that the hepatic inflammation and fibrosis were not independent risk factors associated with RFS or OS, indicating little impact of the hepatic inflammation and fibrosis on the prognosis of HCC patients when receiving continuous antiviral therapy. Only one previous study has used the Scheuer scoring system to explore the role of inflammation and fibrosis in patients with non-cirrhotic HBV-associated HCC. They concluded that hepatic inflammation and fibrosis had a negative impact on the prognosis of HCC [10], which is different from our conclusion possibly because we only included patients with BCLC 0-A stage HCC, while their study included approximately 30% of patients with BCLC stage C HCC, and we included patients with various degree of fibrosis (from minor fibrosis to cirrhosis), but their study excluded patients with cirrhotic liver. Since we consecutively enrolled patients with HBV-related early-stage HCC, it was easy to perform radical therapy and investigate the role of non-tumor factors in the patient’s prognosis. In our study, approximately 53.1% of patients had high HBV-DNA load and 21.0% had positive HBeAg at the time of surgery. This suggests that despite all patients receiving antiviral therapy, the virological management was not satisfactory. The antiviral therapy for all included patients was therefore modulated according to the guidelines preoperatively. Proper management of HBV infection aided to improvement in the histological impairment. Hence, the efficacy of antiviral therapy might reduce the negative impact of underlying liver conditions on the prognosis of HCC.

As a direct index reflecting hepatitis, we found that hepatitis had a close relationship with elevated serum ALT levels and decreased PLT and ALB levels. Notably, severe hepatitis was positively correlated with high HBV-DNA load and a high rate of positive HBeAg. A high proportion of patients with severe hepatitis (68.5%) tended to suffer from liver cirrhosis. This directedly suggests that there was a close relationship between viral status and hepatic inflammation and cirrhosis. Antiviral therapy is critical for HBV-related patients. MVI and satellite lesions are well-known prognostic factors for patients with HCC [19, 20]. Despite no statistical significance, the incidence of MVI and satellite lesions seemed to be higher among patients with severe hepatitis. This suggests that hepatitis can contribute to tumor aggressiveness. In our study, among the HBV-related patients with early-stage HCC, we demonstrated that MVI and satellite lesions were significantly associated with RFS and OS post-hepatectomy. Chronic hepatitis leads to liver fibrosis and cirrhosis. About 59.2% of patients with liver cirrhosis had severe hepatitis, indicating that hepatitis significantly correlates with liver fibrosis. Patients with advanced fibrosis had a significantly high HBV-DNA load and positive HBeAg, reflecting the direct relationship between virological etiology and underlying liver condition. As the fibrosis progressed, the serum PLT level decreased significantly. FIB-4 scoring is an non-invasive method to evaluate liver fibrosis. We found that the FIB-4 scores were significantly higher in patients with cirrhosis. Tumor size was an important prognostic factor for HCC. Herein, we found that advanced liver fibrosis was negatively associated with tumor size. This might be a result of more patients with cirrhosis following regular HCC surveillance. The mechanism of the inverse relationship between tumor size and degree of fibrosis needs further clarification. As reported previously [4, 21], liver cirrhosis might not be a robust prognostic factor associated with HCC recurrence and long-term survival. During the regular postoperative follow-up, good management of HBV infection might contribute to the minimized impact of liver cirrhosis.

The results of the multivariate analysis revealed that tumor-related factors remained a major contributor to the prognosis of patients with early-stage HCC. The prognosis was largely dependent on tumor biology [22]. Except for tumor size, satellite lesion, and MVI, elevated AFP and capsule invasion were related to long-term survival. This was consistent with the results of a previous study [23]. In contrast to the findings of previous studies [24, 25], we found that HBV-DNA or positive HBeAg was not a predictor of the long-term survival of patients. This might be the result of the efficacy of the antiviral therapy. AST was incorporated into some models to predict the prognosis of HCC [26, 27]. It has been demonstrated to be a risk factor associated with RFS and OS in our study. Hepatic inflammation and fibrosis had no statistical correlation with serum AST levels.

There were some limitations in our study. First, this was a single-center and retrospective study. Secondly, since all patients received antiviral therapy, it was difficult to explore the role of antiviral drugs on the prognosis due to the presence of numerous antiviral regimens. During the follow-up period, we did not further evaluate the histological changes and HBV-DNA fluctuation. Third, we only included patients who had HCC caused by HBV; hence, the conclusion might not be suitable for HCC caused by other etiologies.

In conclusion, we found that hepatic inflammation and fibrosis had little impact on the prognosis of HBV-related patients with BCLC stage A HCC who were receiving antiviral therapy. The efficacy of antiviral therapy might maximally alleviate the negative impact of the underlying liver condition on the prognosis.

## Data Availability

The datasets used in the current study are available from the corresponding author on reasonable request.
